# Discovering presence as part of nurse educators’ role modelling at a public nursing college in the North West province

**DOI:** 10.4102/hsag.v26i0.1639

**Published:** 2021-10-29

**Authors:** Tiisetso A. Mofokeng, Emmerentia du Plessis, Kathleen Froneman

**Affiliations:** 1 Department of Nursing, Faculty of Health Sciences, North West College of Nursing, Klerksdorp, South Africa; 2 NuMIQ Research Focus Area, Faculty of Health Sciences, North-West University, Potchefstroom, South Africa

**Keywords:** nurse educator, presence, public nursing college, role model, shadowing

## Abstract

**Background:**

Nursing students learn the science and art of nursing, including presence, from classroom content, using skills in practice, or by watching an experienced nurse interact with a patient. Nursing education must be designed so that nursing students can construct the art and science of nursing practice. Nursing students must be educated to be sound practitioners in the ‘being’ of nursing practice. Nurse educators modelling presence to nursing students will improve the quality of patient care during clinical training and throughout their professional role.

**Aim:**

To explore and describe nurse educators’ role modelling of presence to nursing students.

**Setting:**

This study was conducted at a public nursing college in the North West province.

**Methods:**

A qualitative, ethnographic study was conducted. Purposive sampling was used. Four nurse educators participated in the study and data saturation was reached. Data were collected through shadowing and informal reflective conversations over a period of 8 days.

**Results:**

The following relationships emerged: nurse educators model ‘being professional’, ‘being facilitating, nurturing, caring and compassionate, encouraging, and motivating’, and ‘being purposeful in their nursing education approach’.

**Conclusion:**

Participants role modelled presence to nursing students despite daily challenges in their work.

**Contribution:**

Creating awareness of how nurse educators can model presence despite daily challenges in their work will influence and motivate nursing students to develop presence skills. This will have a positive impact on managing patients in practice. Recommendations can guide nursing education, policy development and future research to strengthen nurse educators modelling presence.

## Introduction

Doctors and nurses spend less time with patients because of the introduction of technology in healthcare, resulting in a greater demand for holistic care, making the act and quality of care more important (Tavernier 2006). Nursing is a holistic, caring discipline that requires nurse educators to teach the art (being present with patients) and science (having the knowledge and skills to care for patients) of nursing. Nursing is an art that entails teaching nursing students how to be sound practitioners in the being of nursing practice, or being present. Presence is the application of the art of nursing (Potter & Frisch 2007) and is an interpersonal process characterised by sensitivity, holism, intimacy, vulnerability and adaptation to unique circumstances (Finfgeld-Connect 2006), during which the patient demonstrates a need for presence and the nurse is willing to offer presence and practice within an environment that is conducive to it. To be consistent with the proffesion’s aims and values of care and compassion, nursing students must understand the importance of presence and be able to develop presence (Gustin & Wagner 2012). Nurse educators’ ability to embody the quality of being there and being with nursing students in all of their humanness by being aware, engaged, responsive, resonant, and supporting and modelling care in a present manner to nursing students is referred to as presence (Bacon 2012; Kleinman 2009).

According to McMahon and Christopher (2011), the importance of relational connection with patients can be emphasised early in the nursing curriculum by introducing and teaching a presence approach. McMahon and Christopher (2011) point out that all levels of education are an opportunity to develop nursing students’ presence skills of ‘being there’ and ‘being with’, which will increase the likelihood that nursing students will be present with patients when providing clinical care. Clinical and academic nurse educators are encouraged to focus on specific areas of role modelling such as opportunities that will help them to identify the need for presence, buffering environmental obstacles to presence, and skilfully assimilating presence whilst attending to other psychomotor tasks to enhance the delivery of presence when planning activities (McMahon & Christopher 2011). A person who acts as a role model serves as an example of the values, attitudes, and behaviours associated with a specific role. Modelling is regarded as a behaviour, an example or success that can be emulated by others, especially younger people such as nursing students (Soanes & Hawker 2013).

Nursing education takes place in universities, and public and private nursing colleges, each with its own set of conditions and approaches to nursing, teaching and role modelling. When looking at public nursing colleges specifically, the challenges they face require a dedicated response from nurse educators. A national audit of public nursing colleges by the South African Department of Health identified the following main challenges affecting public nursing colleges: lack of infrastructure, resource shortages, inaccessibility of clinical facilities because of distance between education and training sites and clinical facilities for practical placement, lack of transport, inadequate number of educators to accompany students, and shortage of nurse accommodation and demonstration rooms (South African Nursing Council 2013). As a result, nurse educators working at a public nursing institution may develop their own approaches to teaching and role modelling presence. As stated by Fetterman (2010), this necessitates a requirement to comprehend and express this social environment from the inside out, particularly when considering the necessity to model presence to nursing students.

### Problem statement

Given the above, it is critical to facilitate the development of presence capacity in nursing students as this establishes the foundations of nursing practice (American Nurses Association 2001). Nursing students learn the art and science of nursing, including presence, in the classroom, through practised skills and repeated interactions with patients or by seeing an experienced nurse interact with a patient (Idczak 2005). Landers et al. (2014) also state that it is important for nurse educators to focus on educational experiences that will help nursing students develop and nurture an in-depth appreciation of the concept of caring, including presence, in their professional practice. Furthermore, in order to preserve the essence of nursing, the components of presence must be defined, refined and measured to enable nurse educators and other nursing leaders to ensure that presence can be taught and modelled effectively (Turpin 2014). Nursing students must be educated in order to be competent practitioners in the field of nursing practice.

Turpin (2014) warns that in the United States, personal attributes which are required to become proficient in presence are no longer a criterion for admittance into the nursing education system, which is becoming into a factory of knowledge workers. Nursing schools’ selection and admission criteria are based on grade point average, nursing grade point average, pre-testing success, and success in science without the evaluation of interpersonal skills. It is possible that the personality and emotional characteristics needed to enable nursing students deliver a holistic care are not present in the candidate pool (Turpin 2014). As stated by McMahon and Christopher (2011), modelling a caring presence and providing structured learning opportunities for nursing students will help them to develop presence skills over time by ‘being with’ and ‘being there’ for their patient during nursing care.

At the onset of this research, it was not known how nurse educators at a public nursing college in the North West province model presence. Public nursing colleges face unique challenges which require nurse educators to respond with dedication and a significant effort to role model the art of nursing, of which presence forms an integral part.

### Research purpose

The purpose of the study was to explore and describe nurse educators’ role modelling of presence to nursing students at the North West province public nursing college.

## Research methodology

### Design

A qualitative, ethnographic design was selected for this study. As stated by Fetterman (2010), ethnography is about telling a credible, rigorous, and authentic story, as it gives voice to people in their own local context, typically relying on verbatim quotations and a ‘thick’ description of events. This design is aimed at understanding views through the eyes of nurse educators, whilst not relying too heavily on theories and concepts prior to the whole investigation in the research (Ejimabo 2015). Saldana (2011) further describes ethnography as the observation and documentation of social life to give an account of a group’s culture. The purpose of this design was to attempt to understand what is happening naturally in the setting and to interpret the data gathered to explain how findings can inform policy, practice or theory.

### Study setting

The study was conducted at one campus of a public nursing college in the North West province and the participants were nurse educators who were involved in the teaching-learning of ± 326 nursing students enrolled in the 4-year diploma in nursing. Shadowing was conducted in the classrooms where nurse educators were conducting classes and, in their offices, when they had interaction with students. Reflective conversations were held in the individual lecturers’ offices before and after classes.

### Sampling process

Purposive sampling was used as this method allowed the researcher to select the participants who could best contribute to the study (Polit & Beck 2021). The planned sample size was between three and five participants, as is typical for ethnographic research (Ferguson 2016), and data saturation was used as a measure to determine the sample size. The actual sample size was four participants, as saturation was reached after the fourth participant. The reason for the small sample size was that rich, dense, in-depth, multifaceted, complex, and comprehensive data were generated through shadowing, field notes and reflective conversations.

For the purpose of this study, the inclusion criteria were the following:

- Nurse educators who have been working at a campus of a public nursing college in the North West province for more than 1 year. This ensured that experienced nurse educators were included in the research and that rich data could be sourced. It also limited the possibility of participants feeling self-conscious and exposed when presenting classes to nursing students during data collection.
- Nurse educators (professional nurses with an additional qualification in nursing education) who are registered with the South African Nursing Council (SANC) as nurse educators. This criterion was included to ensure that appropriate participants were included, and only nurse educators who are legally registered to act as such were included in this research.
- Nurse educators who have been teaching the 4-year diploma in nursing at a public nursing college in the North West province.
- Nurse educators who were willing to be shadowed and to reflect on role modelling presence to nursing students during their interaction with nursing students.

Exclusion criteria included the following:

- Nurse educators who were in clinical facilities during data collection.
- Nurse educators at the college campus where the researcher is employed as a nurse educator, to avoid bias and conflict of interest.
- Nurse educators who have not been working at the public nursing college in the North West province for more than 1 year.

Sampling was done to a point where no new information was obtained and redundancy was achieved after collecting data from four participants (Polit & Beck 2021). Saturation was confirmed by the researcher, supervisor and co-coders.

## Data collection

Data were collected using the following methods:

### Shadowing

The researcher used this technique by closely following a nurse educator over 2 days in different classes, focusing on the role of nurse educators in modelling presence to nursing students (McDonald 2005). Nurse educators were shadowed during a variety of interactions such as class presentations and meeting with small groups or individual nursing students. Gilliat-Ray (2011) describes shadowing as an ethnographic work where the focus is on the daily practice of a single individual, working in a complex institutional social setting, as in the case of this research. These were supplemented by informal reflective conversations with the nurse educators considering the context in which experiences took place (Van der Meide, Olthuis & Leget 2015).

### Informal reflective conversations

The researcher looked at the broad range of themes and question areas to be covered and arranged them in appropriate sequence (Roestenburg et al. 2021). The questions followed a logical sequence and the researcher ensured that they cover the topic thoroughly. Open-ended questions were formulated in such a way that nurse educators could express themselves freely. These questions focused on getting the specific information required for the purpose of the study (Roestenburg et al. 2021). To ensure this, questions were based on the quality of presence as defined by Covington (2003), Bacon (2012) and Kleinman (2009). During the informal reflective conversations, probing questions were based on the data collected during shadowing. As stated by Gray, Grove and Sutherland (2016), this research design provided a framework for studying culture through the insider’s perspective.

### Field notes

The researcher made field notes on what she observed during shadowing, mainly focusing on the nurse educator’s role modelling of behaviour that resembles presence. These are a broader and more interpretive record of events and conversations, representing the observer’s efforts to record information, to synthesise and to understand the data (Polit & Beck 2021). As indicated by Roestenburg et al. (2021), they are written accounts of the things the researcher hears, sees, experiences and thinks about in the course of data collection. Therefore, it is vital for the researcher to make full and accurate notes of what goes on.

### Data analysis

The data were analysed using the steps provided by Creswell and Creswell (2017) for the ethnographic data analysis. The method consisted of ordering the data and organising patterns, categories, and descriptive units and looking for relationships between them. By using these steps, words from the collected data were examined, grouped into meaningful segments, and organised to compare, contrast, and identify patterns that shed light on beliefs and practices of the study participants.

The following processes summarises the approach:

- Organising and preparing data for analysis

This first step refers to transcribing, sorting, and arranging data from different information sources:

- Reading through all the data

The researcher read through the data to get a general sense of the information and overall meaning and to write down general ideas about the data:

- Coding the data

This step involved organising the data into chunks of information and writing a word that represents a category in the margin:

- Description of the setting or people and categories or themes for analysis

The research supervisors also coded the data, and an independent co-coder was included in this process.

### Trustworthiness

Lincoln and Guba’s (1994) five criteria for developing trustworthiness were used during data collection and data analysis and applied as follows. Credibility was ensured by allowing research participants to review, validate and verify the researcher’s interpretations and conclusions (Brink, Van der Walt & Van Rensburg 2018) during the informal reflective conversations by using techniques such as clarification and probing. The researcher used peer review to ensure that the processes and procedures used are acceptable and dependable to adhere to dependability (Brink et al. 2018). Confirmability was ensured by involving co-coders during data analysis (Roestenburg et al. 2021). The researcher made use of data from different sources to collaborate, elaborate and illuminate the research question and more than one data gathering method could be used to strengthen the study’s usefulness for other settings to adhere to transferability (Roestenburg et al. 2021). Authenticity was adhered to by using a co-coder to ensure accurate data analysis and the reporting of findings from the participants’ lived experiences.

### Ethical considerations

Ethical clearance and study approval were obtained from the Health Research Ethics Committee of the Faculty of Health Sciences, North-West University (reference number: NWU-00013-18-S1) and the Department of Health Ethics Committee before commencement of this study. The researcher also obtained permission from the appropriate authorities, such as the Provincial Department of Health and the management of the public nursing college to gain access to the prospective participants. The nurse educators at the research site were provided with all necessary information concerning the study before consent forms were issued. These were signed by all the nurse educators who agreed to participate in this study. Although data was not collected from nursing students, they were part of the observation process and therefore goodwill permission was obtained. Anonymity was maintained by not including any identifying information, ensuring that the collected data cannot be linked to the participants (Polit & Beck 2021), and the data were presented anonymously to the co-coders, namely the research supervisors. As suggested by Gray et al. (2016), the researcher further ensured anonymity by making use of identification codes instead of participants’ names and by keeping names and code numbers separate from the data collected. Confidentiality was ensured by only allowing the independent co-coders to have access to the collected data after signing a confidentiality agreement.

## Results and discussion

Direct quotations from the informal reflective conversations held with the participants and field notes are presented as evidence based on the key concepts of presence.

### Theme 1: Dedication and innovation in a difficult teaching and learning environment

The participants made nursing students feel at ease. This helped the students to participate actively in class. They were given adequate time to ask and respond to questions. The participants worked very hard to create an atmosphere conducive for learning and took all necessary measures to ensure that nursing students were comfortable. This was confirmed during shadowing, the informal reflective conversations as well as the researcher’s field notes.

Large classes made it difficult for nurse educators to be able to identify individual learning needs. In the case of this research, this group of nurse educators seem to have made the decision to embrace the difficult teaching-learning environment to the best of their ability. They demonstrated dedication and perseverance needed for presence to evolve by creating and maintaining a warm and friendly atmosphere.

‘It goes with materials; books must be there. They must read, they must refer.’ (Participant 3, female, 57)

‘What makes it difficult is high numbers of students because you can’t reach all of them.’ (Participant 2, female, 61)

‘Workload is too much, shortage of staff.’ (Participant 2, female, 61)

The literature confirms that environmental characteristics can indeed be thought of as a dynamic force that affects authentic presence and the decisions made by the nurse educator (McMahon & Christopher 2011). Moreover, a caring and supportive learning environment, including enough space, lighting, and ventilation, as well as warmth, support, caring, and trust, is the ideal in nursing education (Froneman, Du Plessis & Koen 2016). As seen in this research, the teaching-learning environment in nursing education is not always ideal (Salminen et al. 2011).

In her study, Ndawo (2012) indicates that even if the nurse educator can be innovative in teaching and learning, the large number of nursing students makes it impossible to use teaching methods that will facilitate meaningful interaction amongst nursing students and thus prevents learning from taking place. The participants coped with challenges related to the large number of students per class to the best of their ability and modelled dedication and innovation as seen from their attempts to make the classes enjoyable to nursing students and to create and maintain a warm, friendly atmosphere.

### Theme 2: Professional educator-student relationship

The participants demonstrated a specific culture regarding their relationship with the nursing students. For instance, they valued a warm and welcoming opening each day. The participants furthermore emphasised that they are in a professional relationship with nursing students, with clear boundaries between the nurse educators and nursing students. Another value that was evident was that nurse educators involved nursing students in decision making in the class by encouraging nursing students to give inputs and accommodating nursing students’ suggestions and requests as much as possible.

All the participants had an open-door policy and the nursing students were encouraged to consult them at any time in relation to their studies. Throughout their interaction with the nursing students, the participants remained professional. The participants valued engaging with and involving all the students, and therefore encouraged teamwork amongst nursing students. Group interaction was promoted by allowing the nursing students to discuss topics as a class, to ensure that nursing students always remain on the same level. The participants corrected nursing students in a manner that would not demotivate or discourage them from participating in the class discussions and encouraged them to consult their nurse educators for assistance any time when they experienced difficulty in their studies, thereby availing themselves by ‘being there’ for the nursing students.

‘You don’t lose focus, nursing students must know that it is not a social relationship.’ (Participant 4, female, 52)

‘I try to be very professional as possible so that when they approach me they should also be professional.’ (Participant 1, female, 62)

‘By understanding my role as an educator, which is my primary role.’ (Participant 3, female, 57)

‘I don’t underestimate my students and that helps me to maintain professionalism.’ (Participant 1, female, 62)

Duffy 2009 (cited by Bacon 2012) states that the quality of presence may best be learned through the caring relationships and modelling of nurse educators during the educational process. Literature also reveals that a positive relationship between the educator and students is much needed in the holistic development of students (Hamre & Pianta, cited by Hussain et al. 2013). When teachers have a positive relationship with students, they influence students’ interest in school and therefore their level of achievement (Da Luz 2015).

### Theme 3: Teaching-learning strategies

The participants clearly adopted a structured approach in their teaching-learning strategies. Lessons were facilitated by means of clear and well-structured presentations. The participants introduced content in an interesting way, by capturing the nursing students’ attention. The participants ensured that there was a clear link between the new content and the content that the nursing students were taught in the previous level. The participants had insight into what they were teaching and they were experts in their subjects. They used this expertise to link theoretical content to real, practical situations that the nursing students encounter when at the clinical facilities, and they gave practical examples. The content was simplified by using authentic examples.

There is a need for flexibility so that the nurse educators can meet the students’ needs. In this study, it was difficult for the participants to model presence completely because of the challenging conditions in which they worked with regard to the amount of content to be covered versus the number of periods allocated for the subject.

‘You must go to their level.’ (Participant 1, female, 62)

‘Good teaching, you must reach your learners, you must not lose them and they must flow with you and give them practical examples.’ (Participant 3, female, 57)

‘Being able to get to students, being able to give them feedback.’ (Participant 2, female, 61)

‘I don’t underestimate my students.’ (Participant 4, female, 52)

McMahon and Christopher (2011) emphasise that modelling presence and providing structured learning opportunities for nursing students will help them to develop presence skills over time.

### Theme 4: Shared values modelled by nurse educators

It was clear that the participants modelled certain values through their behaviour and interaction with the nursing students. They acted professionally throughout, despite the challenging situations in which they work. This was evident from their physical appearance, neat dress code, and their interaction with the nursing students. All the participants came to the class well prepared and displayed expertise.

The participants addressed the nursing students appropriately by using their names and surnames, which was an indication that they have an interest in knowing the nursing students despite the large number of students in class. They continually assessed the nursing students’ understanding and insight into the work during lessons by making use of question and answer sessions between the lessons. During lessons, the participants were able to use humour, which made students feel at ease and relaxed.

‘We are professionals and we must behave and think like professionals in everything we do.’ (Participant 1, female, 62)

‘I become firm and stand my ground, but I become very professional.’ (Participant 2, female, 61)

‘Dress code affects professionalism.’ (Participant 4, female, 52)

‘Thorough preparation is very important; it helps me to maintain professionalism and even my attitude to know that I have different types of students, students must not see a gap of me not knowing my work or not performing well.’ (Participant 2, female, 61)

‘I must prepare thoroughly so that my students can feel that they can rely on me.’ (Participant 3, female, 57)

The values the participants live are also reflected in literature on presence and nursing education. Indeed, caring nurse educators who show concern for nursing students and act as confidants are positive role models and mentors and contribute to the development of the students’ capacity to overcome personal vulnerabilities and environmental adversities (Froneman et al. 2016). Doherty (2016) states that role modelling is a strategy that enables nurse educators to demonstrate their knowledge, skills, and attitudes, thus establishing their role as expert professionals and gaining the respect of nursing students. It makes skills, knowledge, decision making, and professional behaviour accessible to others. As indicated by Curtis and Jensen (2010), possessing adequate experience in the nursing profession is crucial for authentic presence, and the nurse must also have the characteristics of being morally mature.

According to Turpin (2014), common attributes of educator presence include facilitation, monitoring of progress, teaching, interactivity, and assessment. Curtis and Jensen (2010) further state that in nursing, nurses can either be committed to their profession or not, and this commitment can be linked to authentic presence. It is therefore crucial for nurse educators to possess adequate experience in the nursing profession to enable them to be authentically present (Hickman 2013).

### Theme 5: Principles resembling presence that were modelled

The participants instructed the nursing students that it is always very important to treat patients with respect and to use names and titles when addressing patients. The nursing students were encouraged to always be honest with patients by keeping their promises so that patients can trust them, and to improve on how they communicate with other people both at work and in general. The importance of showing courtesy, respect, and dignity when handling patients was emphasised to the nursing students. The participants were polite, understanding, and supportive towards the nursing students. The nursing students were told that they should not be afraid to ask for assistance from peers and nurse educators whenever they experience challenges.

Turpin (2014) indicates that in order to preserve the essence of nursing, the components of presence must be defined, refined, and measured to enable nurse educators to ensure that presence skills can be taught and modelled effectively. Presence is linked with concepts such as caring or with behaviours such as listening and touch (Bright 2015). As stated by Da Luz (2015), caring educators motivate students and thus the learning process is enhanced. Therefore, students feel secure as the environment surrounded by caring educators will allow them to grow and develop their capacities. According to American Nurses Association (ANA 2001), it is critical to facilitate the development of presence capacity in nursing students as this establishes the foundations of nursing practice.

‘By involving students in whatever decisions I make concerning their training.’ (Participant 2, female, 61)

‘Students feel that they are valued.’ (Participant 3, female, 57)

‘The therapeutic use of self.’ (Participant 4, female, 52)

‘I therefore make time for them.’ (Participant 1, female, 62)

A conscious intention of being present in a moment with another person is required, accompanied by the ability and the desire to be present with compassion (Gustin & Wagner 2012). The Nursing students were applauded for their positive responses and the input to boost their morale, and praise was also given for the participation. Difficult situations were handled professionally, calmly, and constructively. Ndawo (2012) indicates that in an enabling learning environment, each student should be viewed as an individual to be valued, cared for, respected, nurtured, understood, and assisted.

### Limitations of the study

This study is limited to one public nursing college in South Africa. However, the findings have wider value as it is embedded in existing literature to show its relevance. Shadowing was the main data collection method for this study. The informal reflective conversations only involved nurse educators who were in class during data collection and therefore limited the findings to the classroom setting.

### Recommendations

It is evident from the findings that nurse educators do model presence to nursing students. However, additional support is required from faculty. The provision of in-service training programmes to create awareness of presence amongst nurse educators is needed to understand the meaning and value of presence in nursing education. Nurse educators should be appreciated for their dedication and innovation in difficult teaching-learning environment. Through the development of professional development programmes, nurse educators can be supported on implementing teaching strategies to instill presence in nursing students who can lead to increase quality patient care.

Management should take a leading role in coaching and supporting nurse educators. Management can support nurse educators by developing policies on modelling presence to nursing students and the implementation of mentoring programmes as additional resources.

Further research is needed to explore different teaching approaches to promote presence skills in nursing education. Research on how presence impacts relational nursing education and relational nursing care is also recommended.

## Conclusion

Factors such as the working environment, the number of students, the amount of work to be covered, and the number of periods allocated per subject created difficulties in terms of modelling presence, because in order for one to be present, there should be flexibility to meet the nursing students’ needs. The participants modelled presence by being dedicated and innovative in the difficult nursing education setting. They maintained a professional educator-student relationship using specific teaching-learning strategies based on shared values and guided by principles that resemble presence.
